# Short communications: a methodological pilot comparing facial, endocrine, and observer-based welfare measures in service dogs (*Canis lupus familiaris*)

**DOI:** 10.1016/j.vas.2026.100903

**Published:** 2026-10-14

**Authors:** Don Cherry, Brittany Florkiewicz, Teddy Lazebnik, Carolyn J. Henry

**Affiliations:** a Research Center for Human–Animal Interaction (ReCHAI), University of Missouri, Columbia, MO, USA; b Department of Psychology, Lyon College, Batesville, AR, USA; c Department of Information Systems, University of Haifa, Haifa, Israel; d Department of Computing, Jönköping University, Jönköping, Sweden; e Lyon College School of Veterinary Medicine, Cabot, AR, USA

**Keywords:** Service dogs, Animal welfare, DogFACS, Cortisol, Behavioral assessment

## Abstract

Service dogs perform complex tasks in cognitively demanding environments, yet their welfare during training is often inferred from performance rather than measured directly. Several behavioral and physiological measures characterize how dogs respond to training, but researchers rarely apply them concurrently to the same animals. For this reason, we compared three such measures in seven purpose-bred Labrador Retrievers from a single litter during standardized early-stage training: facial behaviors associated with negative affective contexts (coded using DogFACS), urinary cortisol, and observer-based behavioral assessments by experienced trainers and non-expert observers. For each measure, dogs were ranked within the cohort according to that measure's directional convention, and all pairings were compared using Kendall's tau (τ). Rankings varied substantially across measures. Expert assessments agreed most strongly with urinary cortisol (τ = 0.67), non-expert assessments agreed with cortisol at a similar level (τ = 0.60), and expert and non-expert assessments agreed strongly with one another (τ = 0.65). By contrast, facial-behavior rankings showed no agreement with cortisol (τ = 0.00) and weak to very weak agreement with the observer measures (τ = 0.25 and 0.14). Because this was a small, closely related cohort assessed without a non-training control condition, these results cannot establish the validity of any measure. They instead show that measures can characterize the same dogs differently, highlighting the methodological challenges of welfare assessment in working dogs and the need for larger, adequately designed studies.

## Highlights

• Three welfare measures were compared in the same early-stage service dogs.
• Cortisol, facial behavior, and observer ratings ranked the same dogs differently.
• Expert and non-expert ratings agreed strongly with each other and with cortisol.
• Facial behavior rankings showed no agreement with cortisol.
• Commonly paired welfare measures may not be interchangeable.

## 1 Introduction

Service dogs (*Canis lupus familiaris*) are trained to perform complex tasks in demanding, unpredictable environments while maintaining focused attention and close coordination with their human partners (Cobb et al., 2021; Helton, 2009). Dogs working in support roles are valued for their contribution to handler well-being, and interaction with dogs more broadly has been associated with benefits across physical, social, and psychological domains (Gee et al., 2017; Handlin et al., 2011; Howell et al., 2022; Odendaal & Meintjes, 2003; Pendry & Vandagriff, 2019). Although much is known about the benefits dogs provide to people, relatively little is known about how the dogs themselves perceive and experience their work (Dawkins, 2012).

This gap is compounded by a more fundamental difficulty: "stress" is not directly observable, as it is a physiological and psychological response to a perceived challenge that can only be inferred from indirect indicators such as endocrine activity, behavior, or expressed affect (Beerda et al., 1997; Mormède et al., 2007). These same indicators also reflect physiological activation, arousal, engagement, or negative affective states that are not specific to stress, yet researchers often treat them as interchangeable. This conflation matters because any inference about stress depends on the biological or behavioral process being measured, the temporal scale over which that process operates, and when the measurement is collected. Each measure provides only a partial snapshot of an animal's response within a particular temporal window, rather than a complete representation of its overall state. Moreover, individual variation in prior experience, rearing environment, and training history further complicates interpretation, as these factors may influence physiological and behavioral responses independently of the immediate context being evaluated. Accounting for both measurement-related and individual sources of variation is therefore important when comparing welfare-related responses across dogs.

Welfare-related responses in dogs are assessed using a broad range of methods, including ethograms quantifying discrete behaviors, cardiac measures such as heart rate and heart rate variability, and indicators of positive affective state such as exploration and play (Beerda et al., 1997; Cobb et al., 2021; van der Staay et al., 2025). This study focuses on three approaches to characterizing welfare-related responses in dogs that differ in both the type of information they capture and the temporal scale over which they operate.

The first involves identifying and quantifying facial muscle movements associated with negative affective contexts, including fear, frustration, and pain (Bremhorst et al., 2022; Caeiro et al., 2017; Mota-Rojas et al., 2021). Although these movements are frequently examined in welfare research, several are context-dependent and may also occur during affiliative, communicative, or attentional interactions, meaning their presence alone does not necessarily indicate a negative affective state (Canori et al., 2025; Capitain et al., 2025; Pedretti et al., 2022).

A second approach involves measuring cortisol, an endocrine indicator of hypothalamic-pituitary-adrenal (HPA) axis activity (Mormède et al., 2007; Sheriff et al., 2011). Cortisol can be quantified using several biological matrices, including blood, saliva, and urine, which differ in their sampling characteristics and temporal resolution. Salivary cortisol is frequently used to examine relatively acute HPA-axis responses, whereas urinary cortisol reflects cortisol secretion pooled over the period preceding urination (Beerda et al., 1996; Möstl & Palme, 2002). In the present study, we selected urinary cortisol because samples could be collected noninvasively without restraint or additional handling, minimizing the possibility that the sampling procedure itself altered physiological activation. This advantage comes with reduced temporal specificity, as urinary cortisol cannot be attributed to a discrete event occurring immediately before sample collection. Accordingly, urinary cortisol was used here to characterize relative physiological activation over the observation period, rather than as a measure of an acute response to the one-hour training session.

The third approach is qualitative behavioral assessment (QBA), in which experienced or naïve observers evaluate a dog's overall behavioral expression using descriptors such as relaxed, confident, or tense (Paul et al., 2005; van der Staay et al., 2025; Wemelsfelder et al., 2001). Rather than focusing on discrete behaviors, QBA captures the expressive quality of behavior as perceived by human observers. These assessments therefore depend on observer interpretation and may vary with an individual's experience, expectations, and familiarity with canine behavior. Human-interpretable descriptors may also introduce anthropomorphic attribution, particularly when perceived behavioral expression is interpreted as a direct representation of the animal's internal state. Observer-based instruments may further combine conceptually distinct features, including perceived affective state, attentional focus, engagement, and responsiveness to cues. Some of these features reflect trained performance as well as current behavioral state, so composite scores are best interpreted as broad summaries of observer perception rather than unitary measures of welfare.

Taken together, these approaches provide different temporal and functional perspectives on the same animal. Facial behavior captures rapid, moment-to-moment changes in behavioral expression, urinary cortisol integrates HPA axis activity over a preceding period, and qualitative behavioral assessment summarizes behavioral expression across a broader observation window (Cohn et al., 2007; Möstl & Palme, 2002). As a result, measurements collected from the same dog during the same training session may characterize that individual differently depending on the process and temporal window being sampled. Agreement among these approaches therefore cannot be assumed, yet few studies have directly compared how measures operating across different biological, behavioral, and temporal scales characterize the same individuals.

Given these methodological differences, the goal of the present study was to compare three approaches used to characterize welfare-related responses in a cohort of dogs enrolled in early-stage service dog training. Specifically, we examined the extent to which urinary cortisol, facial behavior, and qualitative behavioral assessment yielded similar or different characterizations of individuals within the cohort. The observed sessions consisted of foundational obedience, handling, socialization, and environmental exposure rather than role-specific service tasks. This cohort provided a relatively homogeneous population whose members shared age, rearing environment, husbandry, and training curriculum. This shared background reduced variation in several life-history factors that might otherwise complicate comparisons among assessment methods. As a descriptive pilot involving a small and closely related cohort, the study provides an initial comparison of relationships among these assessment methods within this group and is intended to inform the design of future studies evaluating welfare-related responses in broader canine populations.

## 2 Materials and methods

All procedures involving animals were reviewed and approved by the Institutional Animal Care and Use Committee (IACUC) under Protocol 66167 through the University of Missouri College of Veterinary Medicine. Human participant components were deemed exempt under Institutional Review Board (IRB) guidelines (Exemption Category 2), as no identifiable or sensitive information was collected.

### 2.1 Study population

Seven purpose-bred Labrador Retrievers (4 males, 3 females) enrolled in a formal service dog training program at Duo Dogs Inc. (Maryland Heights, Missouri, USA) were included in this study. All seven dogs were from a single litter, approximately six months old at the start of data collection, and shared the same early-rearing environment, husbandry, and training program. At the time of data collection, all dogs were progressing through Stage 1 of training and had not yet begun task-specific placement training. All seven dogs were observed during four weekly training sessions, yielding 28 session-level observations. All sessions were conducted indoors at the training facility under climate-controlled conditions. No elimination breaks occurred during the sessions, and no urine was voided between pre- and post-session sample collection.

#### 2.1.1 Training program description

Stage 1 followed a structured curriculum delivered by the same lead trainer throughout all four weeks. Sessions were held weekly from 5:30 to 6:30 PM in the same designated training space at the facility, where all dogs performed the same set of tasks each week. The curriculum targeted foundational skills common to the AKC Canine Good Citizen framework: basic obedience (sit, down, stay, name recognition, handler focus), impulse control, loose-leash walking, novel-object desensitization, handling and body-manipulation techniques, and exposure to unfamiliar handlers and staff. Each week built on the same core tasks, and every dog completed the same activities in each session, ensuring consistent exposure to tasks, handlers, and environmental stimuli across the cohort.

### 2.2 Video recordings

All training sessions were recorded using two stationary Canon VIXIA HF R800 cameras positioned in opposite corners of the training room and one handheld Panasonic HC-V785 camcorder operated within the room. The Canon cameras remained in fixed positions throughout each session and were positioned consistently across sessions. Observer-based assessments were conducted using the stationary recordings, and all raters reviewed the same full-session footage. Facial behavior coding was conducted from the handheld Panasonic recordings, which provided closer views of individual dogs when the camera operator could capture them clearly. Stationary footage was used to cross-reference behavioral context and confirm dog identity. Handheld filming was opportunistic rather than standardized across dogs.

#### 2.2.1 Facial behavior coding

Facial behaviors were coded using ELAN with a customized annotation template. First, we identified the onset, duration, and offset of all facial behaviors in the selected recordings. These behaviors are defined as one or more facial muscle movements that individuals produce and combine in distinct temporal bouts. Once all facial behaviors were identified, we coded each one according to the protocols established in DogFACS (Waller et al., 2013). We recorded all observed facial muscle movements, including Action Units (AUs) and Action Descriptors (AD), for each facial behavior. In our statistical analyses, we focused on individual facial muscle movements that previous literature has identified as potential indicators of negative affect in dogs, such as those associated with fear, frustration, and pain (see Table 1; Bremhorst et al., 2022; Caeiro et al., 2017; Mota-Rojas et al., 2021). It is important to note that the training regimens involved relatively low levels of physical activity and did not include tasks expected to cause pain. These movements remain context-dependent, however, as canine facial expressions can arise in various social, physiological, and environmental contexts. For example, panting (AD126) may occur in response to thermoregulation, physical exertion, or emotional arousal. Accordingly, we did not interpret any single movement in isolation as evidence of a particular affective state. This approach acknowledges the contextual flexibility of individual facial movements while retaining behaviors repeatedly associated with negative affective contexts in prior studies.

**Table 1:** Facial muscle movements previously associated with negative affective contexts.

Facial Muscle Movement	Description
AU143: Eye Closure	The eyes are closed for >0.5 s.
AU12: Lip Corner Puller	The corners of the lips are drawn back towards the ears.
EAD103: Ear Flattener	The ears are moved backward and flattened against the head, disappearing from frontal view. Visible in floppy ears.
AU109+110: Nose Wrinkler & Upper Lip Raiser	The skin along the muzzle bridge is pulled upward toward the inner corners of the eyes, raising the top lip; teeth may be exposed.
AD126: Panting	Opening the mouth, relaxing the jaw, exposing the tongue, and breathing rapidly.

Coding proceeded on an event basis across all available handheld recordings. Every dog contributed coded events from multiple recordings (range: 12–21 recordings per dog), and the number of coded facial events varied among individuals (range: 50–159). Handheld filming was opportunistic, and the duration for which each dog's face was visible and codable was neither standardized nor quantified. Event counts therefore represent raw frequencies rather than rates and cannot be interpreted independently of differences in observation opportunity. Per-dog recording coverage is reported in the electronic supplement.

Inter-observer reliability was assessed on a random 10% of the video footage prior to full coding. Two measures were used: percentage agreement on the presence of facial behavior events and Wexler's ratio for agreement on the composition of facial muscle movements within each event. Agreement on event presence was 100%, exceeding the conventional 70% criterion. The average Wexler's ratio was 0.703, meeting the 0.70 criterion applied in DogFACS reliability assessment. For within-cohort rankings, dogs exhibiting more of the candidate movements listed in Table 1 received rankings closer to 1, whereas those exhibiting fewer received rankings closer to 7.

#### 2.2.2 Observer-based assessments

Observer-based evaluations used a brief Likert-scale survey assessing perceived calmness, stress, focus, responsiveness to verbal and visual cues, and engagement during sessions (Table 2; Wan et al., 2012). Evaluations were completed independently by five experts (with ≥5 years of professional experience in canine behavioral assessment) and five non-experts (with no formal behavioral education) recruited through a dog boarding and training facility. All raters reviewed the same full-session stationary-camera footage for each dog, were blinded to study aims and handler identity, and identified dogs by collar color to reduce identification bias (Camerlink et al., 2022). No rater had prior familiarity with any of the dogs in the study. Ratings were completed for each dog in each of the four sessions, then averaged across sessions and within each rater group to produce a single composite score per dog.

**Table 2:** Likert-scale items were posed to both experts and non-experts for each dog. Item 2 was reverse-coded so that higher values consistently indicate more favorable overall observer impressions when compiling composite scores.

Item	Question
1	The dog appeared calm and relaxed during the session
2	The dog showed signs of stress during the session (reverse-coded)
3	The dog stayed focused during the session
4	The dog responded promptly to verbal cues during the session
5	The dog responded promptly to visual cues during the session
6	The dog’s engagement level improved during the session

The six survey items captured multiple dimensions of the dogs' observed behavior, including perceived affective state and task performance. Items addressing calmness and signs of stress related most directly to perceived affective state, whereas focus, responsiveness to verbal and visual cues, and engagement reflected aspects of task performance that may also be influenced by training history. Accordingly, the composite was treated as a summary of overall observer impression during the session rather than as a validated index of a single underlying construct. For within-cohort comparison, lower composite scores received rankings closer to 1, reflecting a less favorable overall observer impression, whereas higher composite scores received rankings closer to 7.

#### 2.2.3 Urinary cortisol sampling

Urine samples were collected noninvasively before and after each of the four training sessions using a free-catch method with a telescopic collection device (Hennessy et al., 1997). Handlers collected pre-session samples at home immediately before departing for the facility to minimize potential confounding effects of car travel and arrival at the training environment on baseline cortisol concentrations (Rooney et al., 2007). Handlers transferred these samples to the research team upon arrival, where they were held in an insulated transport cooler with cold packs. After each session, handlers took their dog to void, with up to 15 min allowed for collection. All samples were aliquoted and stored at −80 °C following session completion. Samples were subsequently shipped to the Saint Louis Zoo Endocrinology Laboratory for analysis.

Cortisol was measured using a commercially available enzyme immunoassay (DetectX Cortisol EIA, K003-H; Arbor Assays, Ann Arbor, MI, USA). Prior to assay, 5 µL of β-glucuronidase/arylsulfatase (Roche Diagnostics 10–127–698001) was added to 500 µL of each urine sample and incubated at 37 °C for 24 h. Hydrolyzed samples were diluted 1:100 with assay buffer and assayed according to kit instructions. Creatinine was measured using a commercially available enzyme immunoassay (DetectX Urinary Creatinine EIA, K002-H; Arbor Assays), with samples diluted 1:20 in deionized water. Cortisol concentrations wer**e** normalized to creatinine concentration (ng/mg Cr) to account for urine dilution (Weber et al., 2010). All samples were assayed in duplicate in a single assay for each analyte; mean intra-assay coefficients of variation were 8.9% for cortisol and 7.8% for creatinine. Because we ran only one assay per analyte, we did not calculate inter-assay variation. Assay validation in canine urine demonstrated parallelism across serial dilutions (1:2, 1:4, 1:8) of five high-concentration samples, recovering 101.5 ± 1.4% of expected values, and accuracy on spike recovery at two dosage levels in five low-concentration samples, recovering 100.4 ± 1.3% of added cortisol.

Complete pre- and post-session data were available for five of the seven dogs; the remaining two did not provide sufficient samples and were therefore not assigned cortisol rankings. Pairwise cortisol comparisons were therefore based on these five dogs, while comparisons among the remaining measures used all seven.

Sample timing was considered when determining how urinary cortisol values were summarized. Maximum urinary cortisol concentration has been reported approximately three hours after a stressor in dogs (Rooney et al., 2007), whereas post-session samples in the present study were collected within 15 min of the session ending. These samples therefore cannot be attributed specifically to the preceding training session and likely reflect cortisol secretion accumulated over a broader preceding period (Möstl & Palme, 2002). Using post-session values alone would consequently imply a degree of temporal specificity that urinary cortisol does not provide. Across the 20 available pre- and post-session comparisons, cortisol increased in 11 and decreased in nine, with no consistent direction across dogs or sessions. We therefore summarized each dog with a single mean concentration across all available samples, treating this as an index of relative physiological activation across the observation period rather than as a response to any individual session. Session-level values for all dogs are reported in the electronic supplement, and dogs with higher mean cortisol received rankings closer to 1.

### 2.3 Data analysis

Each measure produced a single within-cohort ranking for each dog. To ensure comparability, all measures were oriented using the same convention: a ranking of 1 was assigned to the dog showing the highest level of that measure's putative welfare-relevant response (i.e., the most negative-affect facial behaviors, the highest mean cortisol, or the lowest observer composite score). This shared orientation means that positive τ values indicate that two measures ordered the cohort in the same direction. We did not assume that this ordering reflects the true severity of stress; it is a descriptive device for comparing measures. Rankings are relative to the cohort by construction: a dog ranked 1 on a given measure scored highest within this group of seven, which carries no implication about absolute level.

Rankings derived from facial behavior, expert observer assessments, non-expert observer assessments, and urinary cortisol were compared pairwise using Kendall's tau (τ), a nonparametric measure of rank agreement. Ties were assigned the same rank. We report τ magnitudes using standard descriptive bands (0.00–0.20 = very weak, 0.21–0.40 = weak, 0.41–0.60 = moderate, 0.61–0.80 = strong, 0.81–1.00 = very strong). Given the small cohort (five dogs for any pairing involving cortisol, seven otherwise), these values are descriptive only and should not be interpreted as hypothesis tests. With cohorts this small, τ is highly sensitive to individual placement, such that small changes in rank order can meaningfully alter τ and may move a value between descriptive bands. As a result, the bands should be read as rough labels rather than as boundaries with substantive meaning.

## 3 Results

Counts of facial behaviors associated with negative affective contexts ranged from 25 to 146 across dogs (median = 60). Expert composite scores ranged from 16.00 to 28.00 (median = 23.00), and non-expert composite scores ranged from 21.75 to 26.25 (median = 24.25). Mean urinary cortisol ranged from 65.11 to 137.59 ng/mg Cr across the five dogs with complete data, and individual creatinine-corrected samples ranged from 39.77 to 308.69 ng/mg Cr. Within-cohort rankings for all four measures are summarized in Table 3; full dog-level descriptive values are provided in the electronic supplement.

**Table 3:** Within-cohort rankings for each dog across the four measures. Two dogs were not assigned due to insufficient urine samples.

Dog Name	Cortisol Rank	Facial Rank	Expert Rank	Non-Expert Rank
Dog 1	1	7	4	4
Dog 2	N/A	5	3	6
Dog 3	5	3	5	7
Dog 4	4	6	5	5
Dog 5	2	1	2	2
Dog 6	3	2	4	3
Dog 7	N/A	4	1	1

Pairwise rank agreement ranged from no agreement to strong agreement (Fig. 1). Agreement was strongest between expert observer assessments and cortisol rankings (τ = 0.67) and between non-expert assessments and cortisol rankings (τ = 0.60), with strong agreement between expert and non-expert assessments (τ = 0.65). By contrast, facial-behavior rankings showed no agreement with cortisol (τ = 0.00), weak agreement with expert assessments (τ = 0.25), and very weak agreement with non-expert assessments (τ = 0.14).

**Fig. 1:**
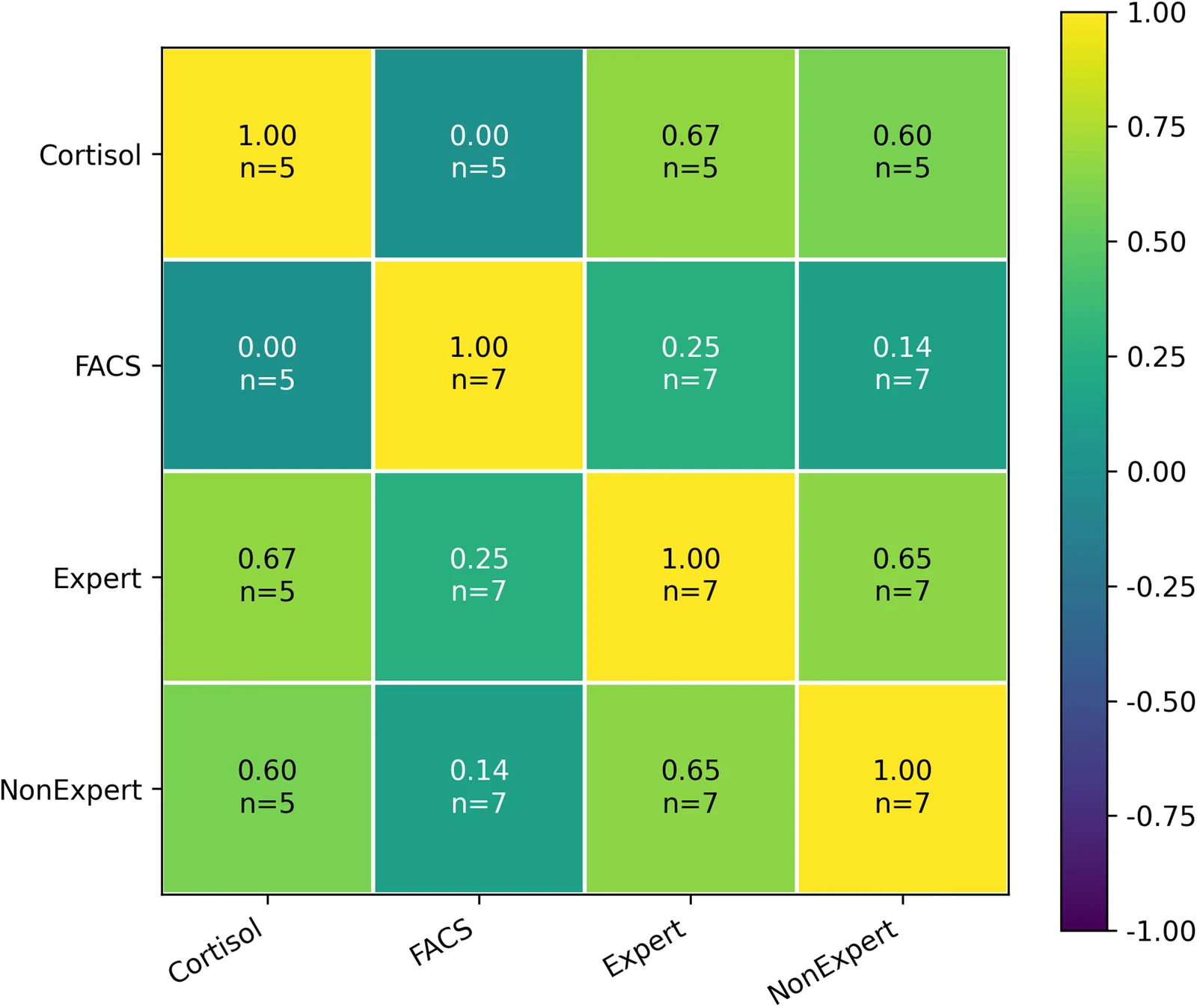
Heat map of Kendall rank agreements.

## 4 Discussion

The present descriptive pilot compared how three approaches to assessing welfare-related responses characterized the same cohort of dogs during standardized early-stage training. Across the cohort, urinary cortisol, facial behaviors, and observer-based assessments did not consistently characterize individual dogs. Observer-based assessments showed strong agreement with one another and with averaged urinary cortisol values, whereas facial behavior rankings differed substantially from both. Collectively, these findings indicate that assessment methods may yield different characterizations of the same individuals, even when observations are collected from the same dogs during the same training period. This is a methodological observation about how these measures relate to one another; it does not indicate that training affected welfare in any particular direction, which the present design was not built to assess.

One possible explanation for the differing patterns observed across measures relates to the structure of Stage 1 training. Dogs were required to sustain engagement and task performance for approximately one hour of training while encountering multiple exercises and environmental challenges. Under these conditions, overall behavioral presentation may have remained relatively stable, yielding similar observer-based assessments, whereas facial expressions fluctuated in response to individual moments within the session and urinary cortisol reflected physiological activation pooled across a broader preceding window. Consequently, the differing patterns observed across measures may reflect the distinct aspects of the training experience captured by each method rather than conflicting assessments of the dogs themselves.

A comparable divergence between behavioral and physiological measures has been reported in dogs. Rooney et al. (2007) followed 31 male Labrador Retrievers of similar age and rearing history through introduction to kennels and found no significant correlations between any behavioral variable and urinary cortisol/creatinine ratio across the population, despite the sample's homogeneity. They concluded that urinary cortisol/creatinine ratio responded to the imposed stressor more predictably than behavioral measures did, while cautioning against exclusive reliance on any single welfare indicator. These findings align with the broader observation that behavioral and physiological measures need not covary, though this descriptive pilot provides no basis for privileging one measure over another. With five dogs contributing complete cortisol data and seven contributing behavioral and observer-based data, these results instead illustrate the methodological consequences of treating different welfare-related measures as interchangeable.

The differing patterns observed across assessment methods highlight an important challenge in evaluating welfare in working dogs. No single measure captures every aspect of an animal's response during training. Physiological measures, facial behavior, and observer-based assessments each provide distinct perspectives on canine responses and are influenced by their own assumptions and methodological constraints. Consequently, agreement among measures should not necessarily be expected in all circumstances, nor should disagreement be viewed as evidence that one approach is inherently superior to another. Instead, these findings emphasize the importance of interpreting each assessment within the context of what it measures and the temporal window over which that information is collected.

### 4.1 Limitations

The present findings should also be interpreted in light of several important methodological limitations. First, pre-session samples collected at home provided a baseline measure prior to training, but the study lacked a non-training control condition. Therefore, training-related changes could not be distinguished from physiological variation that may occur across the same period on a non-training day. Second, urinary cortisol values were averaged across pre- and post-training samples, limiting interpretation of acute within-session physiological responses. Third, all dogs originated from the same litter, shared early developmental experiences, and completed the same training program. This homogeneity was advantageous for a methodological comparison, since it limits the influence of breed, age, and rearing differences on how the measures ordered individuals. However, it also reduces the independence of observations and limits generalizability beyond this cohort. The urinary cortisol dataset was further reduced by incomplete sample collection, and facial behavior frequencies were not normalized for visible face time or observation opportunity. Another consideration is that the observer composite combined items addressing perceived affective state with items addressing task performance, so its agreement with cortisol is harder to interpret than agreement between two measures of a single construct. Finally, observations were limited to four weekly training sessions during Stage 1 of training and therefore represent only a brief developmental period. Because the measures only rank dogs relative to one another, the highest- and lowest-ranked dogs will always emerge, even if none experienced meaningful stress. With cohorts of five to seven, these rankings are also sensitive to individual dog placement. Therefore, the rankings should not be read as absolute levels. Collectively, these considerations emphasize that the present findings should be interpreted as descriptive observations.

Despite these limitations, the present study offers a preliminary methodological comparison of three assessment approaches used in canine welfare research. By concurrently evaluating urinary cortisol, facial behavior, and observer-based assessments in the same dogs, this study highlights the challenges of comparing measures that differ in both temporal resolution and the type of information they capture. These findings provide a foundation for future studies designed to evaluate how welfare assessment methods relate under more controlled experimental conditions.

Future work should incorporate baseline and non-training control sampling, analyze physiological responses at the session level rather than using aggregated measures, include larger cohorts of unrelated dogs across multiple training programs, and provide more detailed descriptions of training activities and environmental exposures. Coding facial behavior outside the training context would establish baseline rates of individual facial movements for each dog, allowing training-session behavior to be evaluated relative to that individual's typical expression. Standardizing or quantifying codable face time would further allow facial-event frequencies to be expressed as rates, improving comparisons among individuals with different observation opportunities. Mixed-effects models accounting for handler, session, and observation opportunity would also make better use of session-level data than the aggregated approach used here. Additional physiological measures, such as heart rate variability, could help determine whether differences among measures reflect the responses they capture or methodological differences in how those responses are sampled. Continued refinement of facial behavior coding, together with advances in automated behavioral analysis and wearable technologies, may further improve understanding of how different assessment methods characterize welfare-related responses throughout service dog training.

## Ethics declaration

Animal subject

This study was conducted in accordance with the ARRIVE (Animal Research: Reporting of In Vivo Experiments) guidelines.

This study was approved by the ACUC.

(Approval No. 66167)

## CRediT authorship contribution statement

**Don Cherry:** Writing – review & editing, Writing – original draft, Visualization, Methodology, Investigation, Formal analysis, Data curation, Conceptualization. **Brittany Florkiewicz:** Writing – review & editing, Writing – original draft, Supervision, Project administration, Methodology, Conceptualization. **Teddy Lazebnik:** Writing – review & editing, Validation, Software, Methodology, Formal analysis, Data curation. **Carolyn J. Henry:** Writing – review & editing, Validation, Supervision, Project administration.

## Declaration of competing interest

The authors declare no known competing financial interests or personal relationships that could have influenced the work reported in this paper.

## Data Availability

The datasets generated and analyzed during the current study are available from the corresponding author upon reasonable request. Due to ethical and privacy considerations associated with video recordings of handlers and training sessions, raw video data are not publicly available. Edited video clips used for illustrative purposes in this publication were included with informed consent from all participating individuals and organizations.
