# Supplementary material for: Short communications: a methodological pilot comparing facial, endocrine, and observer-based welfare measures in service dogs (Canis lupus familiaris)
Source: Vet Anim Sci. 2026 Oct 14;34:100903. doi: 10.1016/j.vas.2026.100903 (PMC13696180; doi:10.1016/j.vas.2026.100903)
Supplement: Supplementary file 1 [file mmc2.docx]

**Electronic Supplement For:**

*Short Communications: A Methodological Pilot Comparing Facial, Endocrine, and Observer-Based Welfare Measures in Service Dogs (Canis lupus familiaris)*

**PART A: SUPPLEMENTARY TABLES**

Table S1. Frequency of the five candidate facial muscle movements associated with negative affective contexts, recorded across all seven dogs. Counts include every coded facial behavior event in which the movement was present, whether alone or in combination with other action units.

| **Dog** | **AU143** | **AU12** | **EAD103** | **AU109+110** | **AD126** | **TOTAL** |
| --- | --- | --- | --- | --- | --- | --- |
| Dog 1 | 3 | 20 | 1 | 0 | 1 | 25 |
| Dog 2 | 4 | 28 | 3 | 2 | 14 | 51 |
| Dog 3 | 3 | 52 | 6 | 1 | 2 | 64 |
| Dog 4 | 3 | 16 | 5 | 1 | 4 | 29 |
| Dog 5 | 31 | 81 | 19 | 5 | 10 | 146 |
| Dog 6 | 11 | 55 | 6 | 2 | 3 | 77 |
| Dog 7 | 9 | 36 | 5 | 1 | 9 | 60 |

Table S2. Facial behavior coding coverage per dog. Recordings indicates the number of distinct handheld video files in which the dog was coded; coded events indicates the total number of facial behavior events annotated for that dog across all recordings; coded duration is the summed duration of those events. Because handheld filming was opportunistic, these values reflect observation opportunity as well as behavioral expression and are not a measure of total visible face time.

| **Dog** | **Recordings** | **Coded events** | **Coded duration (s)** |
| --- | --- | --- | --- |
| Dog 1 | 16 | 101 | 135.8 |
| Dog 2 | 17 | 50 | 58.6 |
| Dog 3 | 17 | 120 | 152.5 |
| Dog 4 | 14 | 70 | 76.8 |
| Dog 5 | 20 | 159 | 141.3 |
| Dog 6 | 12 | 84 | 72.7 |
| Dog 7 | 21 | 84 | 101.9 |

Table S3. Average expert survey ratings across behavioral assessment categories for all seven dogs. Values represent mean Likert-scale scores across trainer evaluations. Stress values were reverse-coded prior to analysis.

| **Dog** | **Calm** | **Stress** | **Focus** | **Verbal** | **Visual** | **Engagement** | **Total Score** |
| --- | --- | --- | --- | --- | --- | --- | --- |
| Dog 1 | 4.25 | 3.25 | 3.50 | 4.00 | 4.00 | 4.00 | 23.00 |
| Dog 2 | 3.00 | 3.25 | 3.25 | 3.75 | 3.75 | 3.75 | 20.75 |
| Dog 3 | 5.00 | 4.50 | 4.75 | 5.00 | 4.50 | 4.25 | 28.00 |
| Dog 4 | 4.75 | 4.75 | 4.50 | 4.75 | 4.75 | 4.50 | 28.00 |
| Dog 5 | 2.50 | 2.75 | 2.75 | 3.00 | 2.25 | 3.00 | 16.25 |
| Dog 6 | 3.50 | 3.50 | 3.50 | 4.50 | 4.25 | 3.75 | 23.00 |
| Dog 7 | 2.50 | 3.00 | 2.75 | 2.50 | 2.25 | 3.00 | 16.00 |

Table S4. Average non-expert survey ratings across behavioral assessment categories for all seven dogs. Values represent mean Likert-scale scores across observer evaluations. Stress values were reverse-coded prior to analysis.

| **Dog** | **Calm** | **Stress** | **Focus** | **Verbal** | **Visual** | **Engagement** | **Total Score** |
| --- | --- | --- | --- | --- | --- | --- | --- |
| Dog 1 | 4.00 | 3.00 | 4.25 | 4.25 | 4.25 | 4.50 | 24.25 |
| Dog 2 | 4.25 | 4.25 | 4.25 | 4.25 | 4.25 | 4.00 | 25.25 |
| Dog 3 | 4.50 | 3.75 | 4.50 | 4.50 | 4.50 | 4.50 | 26.25 |
| Dog 4 | 4.50 | 3.50 | 4.00 | 4.25 | 4.25 | 4.50 | 25.00 |
| Dog 5 | 3.75 | 3.00 | 3.75 | 4.00 | 4.00 | 4.25 | 22.75 |
| Dog 6 | 4.75 | 3.00 | 4.50 | 4.00 | 4.00 | 3.50 | 23.75 |
| Dog 7 | 3.75 | 3.00 | 3.50 | 3.75 | 3.50 | 4.25 | 21.75 |

Table S5. Urinary cortisol-to-creatinine ratio (UCCR; ng/mg Cr) values collected before and after each weekly training session for the five dogs with complete urinary cortisol data. Average values represent the mean cortisol concentration across all available samples. Dog numbering is consistent with Tables S1–S4; Dogs 2 and 7 did not provide sufficient samples.

| **Dog** | **Week 1 PRE** | **Week 1 POST** | **Week 2 PRE** | **Week 2 POST** | **Week 3 PRE** | **Week 3 POST** | **Week 4 PRE** | **Week 4 POST** | **Average** |
| --- | --- | --- | --- | --- | --- | --- | --- | --- | --- |
| Dog 1 | 124.68 | 241.60 | 90.21 | 308.69 | 87.07 | 75.58 | 96.37 | 76.51 | 137.59 |
| Dog 3 | 74.93 | 87.05 | 46.54 | 50.17 | 56.54 | 70.76 | 90.45 | 44.44 | 65.11 |
| Dog 4 | 153.65 | 66.11 | 67.83 | 56.02 | 55.65 | 57.99 | 42.03 | 39.77 | 67.38 |
| Dog 5 | 140.77 | 123.48 | 153.15 | 97.03 | 167.53 | 93.62 | 97.13 | 135.14 | 125.98 |
| Dog 6 | 109.32 | 162.59 | 91.10 | 108.51 | 111.32 | 153.02 | 80.87 | 93.51 | 113.78 |

**PART B: SUPPLEMENTARY INFORMATION**

DogFACS inter-observer reliability. Prior to full coding, a random sample comprising 10% of the video footage was selected for inter-observer reliability (IOR) assessment, consistent with previous facial coding reliability procedures. Inter-observer reliability was evaluated based on (1) agreement on the presence of facial behavior events and (2) agreement on the composition of facial muscle movements within each event. Agreement on the presence of facial behavior events was assessed using percentage agreement, with values at or above 70% considered acceptable. Agreement on facial behavior composition was assessed using Wexler’s ratio, consistent with established FACS reliability methodologies and DogFACS certification standards (Camerlink et al., 2022). An average Wexler’s ratio at or above 0.70 was considered indicative of acceptable inter-observer agreement. In the present study, agreement on the presence of facial behavior events was 100%, and the average Wexler’s ratio for facial muscle composition was 0.703, indicating acceptable reliability.

Derivation of facial behavior counts. Coders annotated all observable facial action units for each facial behavior event, together with concurrent contextual information. The counts reported in Table S1 were extracted from this complete annotation set by identifying, for each event, whether each of the five candidate movements listed in Table 1 of the main text was present. A movement was counted whether it occurred alone or as part of a multi-unit combination within the same event. The complete event-level annotation set is provided in the accompanying Excel workbook.

**PART C: SUPPLEMENTARY FILE DESCRIPTIONS**

The following Excel workbook is provided as a supplementary data file accompanying this manuscript: Comparing Welfare Measures in Service Dogs Supplement A. This workbook contains the complete raw and processed data used in all analyses reported in the present study and is organized across the following sheets:

**FACS Event Data —** Complete event-level DogFACS annotation set, including start and end time, duration, dog identity, session type, gaze fixation, positional behavior, human interaction, and all coded facial action units for each facial behavior event.

**FACS Summary —** Per-dog counts of the five candidate facial movements and coding coverage, as reported in Tables S1 and S2.

**Cortisol Results —** Raw urinary cortisol-to-creatinine ratio (UCCR; ng/mg Cr) values collected before and after each of the four weekly training sessions for the five dogs with complete data.

**Average Expert —** Mean Likert ratings per survey item averaged across expert observers for each dog.

**Average Likert Non-Expert —** Mean Likert ratings per survey item averaged across non-expert observers for each dog.

**Trainer Survey: [Dog] —** Week-by-week raw Likert ratings from expert trainers for each individual dog (one sheet per dog, seven sheets total).

**Spectator Survey: [Dog] —** Week-by-week raw Likert ratings from non-expert observers for each individual dog (one sheet per dog, seven sheets total).
